# The dual role of circHIPK3 in cancer and its implications for multiple drugs resistance: a systematic review and computational approach

**DOI:** 10.3389/fonc.2025.1547889

**Published:** 2025-02-21

**Authors:** Marcelo Monteiro Campelo, Laís Reis-das-Mercês, Amanda Ferreira Vidal, Felipe Rodolfo Pereira da Silva, Ana Carolina Alves de Oliveira, José Rogério de Souza Monteiro, Caique Guimarães Cabral, Renata Coelho Rodrigues Noronha, Adenilson Leão Pereira

**Affiliations:** ^1^ Laboratory of Genetics and Evidence-Based Medicine, Faculty of Medicine, Federal University of Pará, Altamira, Pará, Brazil; ^2^ Laboratory of Human and Medical Genetics, Institute of Biological Sciences, Graduate Program of Genetics and Molecular Biology, Federal University of Pará, Belém, Pará, Brazil; ^3^ Vale Institute of Technology, Belém, Pará, Brazil; ^4^ Graduate Programin Biodiversity and Conservation, Federal University of Pará, Altamira, Pará, Brazil; ^5^ Laboratory of Genetics and Cell Biology, Centro de Estudos Avançados da Biodiversidade (CEABIO), Federal University of Pará, Belém, Pará, Brazil

**Keywords:** circular RNA, CircHIPK3, RBP, microRNA, bladder cancer, chemoresistance

## Abstract

**Background:**

circHIPK3 role in cancer as oncogene or tumor suppressor is still debated, therefore, this study aimed to understand the dual role of this circRNA in different cancers. Furthermore, all available evidence of circHIPK3 interactions with sponged-miRNA and RBPs in oncological diseases were systematically gathered to better understand the its functional role in cancer.

**Methods:**

PubMed, BioMedCentral, Web of Science, Embase and Scopus databases were searched for articles published until October 2024, following the PRISMA guideline. In computational analysis, miRNAs’ sponged target genes and RBPs were used for gene enrichment in KEGG, REACTOME and Gene Ontology, and TISSUES expression. miRTargetLink 2.0 was used to search for target genes, and STRING v.12.0 for gene enrichment.

**Results:**

circHIPK3 can regulate 33 miRNAs which regulate 399 target genes, and that were mainly enriched in major biological pathways important for cancer development and promoting. circHIPK3/miR-124-3p/miR-637/miR-338-3p are the most well documented interactions in cancers that may control MAPK, Jak/STAT3, Wnt/β-catenin, and PI3K/Akt signaling pathways. circHIPK3 regulates miRNAs that modulate genes responsible for chemoresistance, such as ATP-binding cassette and solute carrier transporters genes, and DNA repair genes. circHIPK3 has binding sites for RBPs, which participate mainly of RNA processing and control, and gene expression regulation. Finally, we believe that it has an onco-circRNA role in most cancers, except in bladder cancer, where it has a TS-circRNA function likely due to the microenvironment permeated by high amounts of hydrogen peroxide.

**Conclusion:**

circHIPK3 dysregulation is an important mechanism for cancer establishment, progression and chemoresistance making it an interesting molecule with a potential therapeutic target.

## 1 Introduction

Bladder, breast, cervix and corpus uteri, colorectum, leukemia, liver, lung, esophagus, ovary, pancreas, prostate, stomach, and thyroid cancers stand out among the types of tumors with the highest incidence and mortality in the world population (1). Integrative genomic studies and protein analysis have characterized and identified a complex but not fully understood interaction network involved in tumors’ molecular pathogenesis, such as chromosomal instability, microsatellite instability, hypermethylation phenotypes, gene mutation, non-coding RNA (ncRNA), and protein aberrant expression (2).

A series of recent studies have reported in cancer the dysregulation of a new class of ncRNA, called circular RNA (circRNA) (3). CircRNAs are classified as long ncRNAs due to their >200 nt length, and are derived from pre-mRNA that have their 5’ and 3’ ends covalently joined in a closed loop structure through backsplicing (4). Among the hundreds of dysregulated circRNAs in cancer, circHIPK3 stands out as one of the most reported in many types (3, 5–73). circHIPK3 or hsa_circ_0000284 is derived from exon 2 (1,099 bp) of the Homeodomain Interacting Protein Kinase 3 (*HIPK3*) gene, located on human chromosome 11 (chr11:33278868-33378568) (5).

This circRNA was described acting as both RNA-binding proteins (RBP) and miRNA sponge (3, 5–73) in different types of cancers, but the miRNA sponge function is the most explored and understood one (3, 5, 8, 11–24, 26–28, 33–58, 60, 62, 64–73). For instance, one of the first reports for circHIPK3 found its overexpression in cancer cells and identified at least 18 binding sites for 9 different miRNAs; it was also noticed that it acts as a modulator of cancer cell growth by sponging *miR-124-3p* (5). In addition, circHIPK3 was predicted to have binding sites for 50 different RBPs (3, 30).

Several studies on different types of cancers reported the upregulation of circHIPK3 and its important role as a miRNAs’ sponge contributing to cancer onset and development. In blood (6, 23), bone (60), breast (11–16), cerebral (38–42), gastrointestinal (5, 19–22, 24–30, 32–36, 43–47, 64, 65), gynecological (17, 18, 61, 62), head and neck (55–58), kidney (72, 73), lung (48–53), prostate (66–70), and skin (54) cancers it is overexpressed, and is able to promote cell proliferation, migration, invasion, apoptosis inhibition, metastasis, and chemoresistance. For example, its overexpression can affect the axes *miR-193a-5p*/*HMGB1/PI3K/AKT* in breast cancer (11), *miR-338-3p/HIF-1α* in cervical (17), *miR-637/STAT3/Bcl-2/beclin1* in colorectal (19), *miR-124/B4GALT1*, NF-κB in chronic myeloid leukemia (23), *miR-124-3p/STAT3* in glioma (38), *miR-124/SphK1*/*STAT3*/*CDK4* in lung (48), *miR-215–5p/YY1* in melanoma (54) and *miR-7/VEGF* in ovarian (62), contributing to cancer cell growth, proliferation, invasion, migration, epithelial-mesenchymal transitions, apoptosis inhibition, and chemoresistance.

The under expression of circHIPK3 has also been reported in some studies (31, 59, 63, 71), mainly for bladder cancer (7–10). For example, its under expression can affect the *miR-588/HPSE* axis favoring cell migration, invasion, and angiogenesis (8). Its reduced expression is related to progression (7) and gemcitabine resistance in bladder cancer (9), and it can negatively regulate autophagy (10).

The dysregulation of circHIPK3 is undoubtedly an important mechanism for cancer development. However, its biological role as oncogenic (onco-circ) and/or tumor suppressor (TS-circ) among the different types of tumors is not clear yet and well established. In most cancers, circHIPK3 is upregulated (5, 6, 11–30, 32–58, 60–62, 64–70, 72, 73), but it is known to be downregulated in bladder cancer (7–10). Therefore, the careful searching for new pathways that converge to different tumors may improve the understanding of the mechanisms involved in the pathogenesis mediated by this circRNA.

Here, we gathered all available evidence associated with the mRNA-miRNA-circHIPK3-RBPs axis in oncological diseases in search of its functional role in cancer. The evidence demonstrates that it has a wide onco-circRNA role in cancer, except in bladder cancer, in which it likely has a H_2_O_2_-dependent TS-circRNA function. Furthermore, the dysregulation of this molecule is an important mechanism for resistance to a broad spectrum of chemotherapy drugs. In this scenario, these data open new perspectives towards its use as a potential therapeutic target in cancers.

## 2 Methods

### 2.1 Protocols and registration

We followed the Preferred Reporting Items for Systematic Reviews and Meta-Analyses (PRISMA) guideline to perform this study (74). This study is currently submitted to PROSPERO, under submission number: 628708.

### 2.2 Study design

This study is a systematic review with computational analysis on the role of circHIPK3 in cancer. This study does not require approval by Ethic Committee.

### 2.3 Searching strategy for circHIPK3 in cancer

#### 2.3.1 Inclusion and exclusion criteria

We selected only original studies that conducted experimental validation by “strong evidence” (e.g., RT-qPCR, western blot, luciferase reporter and/or cell assay) of circHIPK3 in human cancer. Papers based on computational prediction analysis without experimental validation or retracted were excluded. No language restriction was applied in the search.

#### 2.3.2 Searching strategy for circHIPK3 in human cancers

A literature search was performed for studies reporting circHIPK3 in cancers in PubMed, BioMedCentral, Web of science, Embase and Scopus databases. The search was conducted until October 1, 2024, in which we used the following entries: “circHIPK3 OR hsa_circ_0000284” and “cancer” combined with boolean operators.

#### 2.3.3 Selection process

For eligibility, two independent reviewers (M.M.C. and L.R-das-M.) screened all records provided by the databases. Titles and abstracts were reviewed, followed by full-text reviews of potentially eligible studies. Review disagreements were resolved through consensus among reviewers and/or consultation with a third reviewer (A.L.P.).

#### 2.3.4 Data collection process

Two reviewers (M.M.C. and L.R-das-M.) independently extracted data from the included studies using a data extraction form that was provided as **Supplementary Material**. Data were collected on the cancer type, sample type and size, circHIPK3 expression profile, sponged miRNAs, experimental validation methods, country of origin, biological function and/or clinical significance, first author and year of publication. Extraction disagreements were resolved through consensus among reviewers and/or consultation with a third reviewer (A.L.P.).

### 2.4 Computational analysis

#### 2.4.1 Searching for target genes of the sponged miRNAs and circHIPK3/RBPs interactions

To understand the functional role of circHIPK3, its interactions with miRNAs and RBPs was explored. At first, sponged miRNAs were collected from the selected studies, and their target genes were found through miRTargetLink 2.0 tool (https://ccb-compute.cs.uni-saarland.de/mirtargetlink2/) (75). This tool was chosen among many others because it is directly connected to the miRTarBase database (http://miRTarBase.cuhk.edu.cn/) (76), which provides information on experimentally validated interaction between the miRNA and its target genes.

In miRTargetLink 2.0, only target genes whose interaction with their target miRNA was experimentally validated by “strong evidence” (e.g., RT-qPCR, western blot, luciferase reporter and/or cell assay) were included in the analyses. The RBPs that were used in functional enrichment, were obtained from previous studies that predicted the circHIPK3-RBPs interactions (3, 30).

#### 2.4.2 RBPs and target gene enrichment analysis

miRNAs-target genes and RBPs were used to perform functional analyses in Kyoto Encyclopedia of Genes and Genomes – KEGG (https://www.genome.jp/kegg/), Reactome (https://reactome.org/) pathways, Gene Ontology – GO (http://geneontology.org/), and TISSUES expression database 2.0 (https://tissues.jensenlab.org) through STRING: functional protein association networks v.12.0 tool (https://string-db.org/) (77). For the statistical significance of gene enrichment, STRING v.12.0 default was applied for p-value adjustment to false discovery rate (FDR_adj_), using Benjamini-Hochberg correction method, and FDR_adj_<0.05.

## 3 Results

### 3.1 Study search strategy

**Figure 1:**
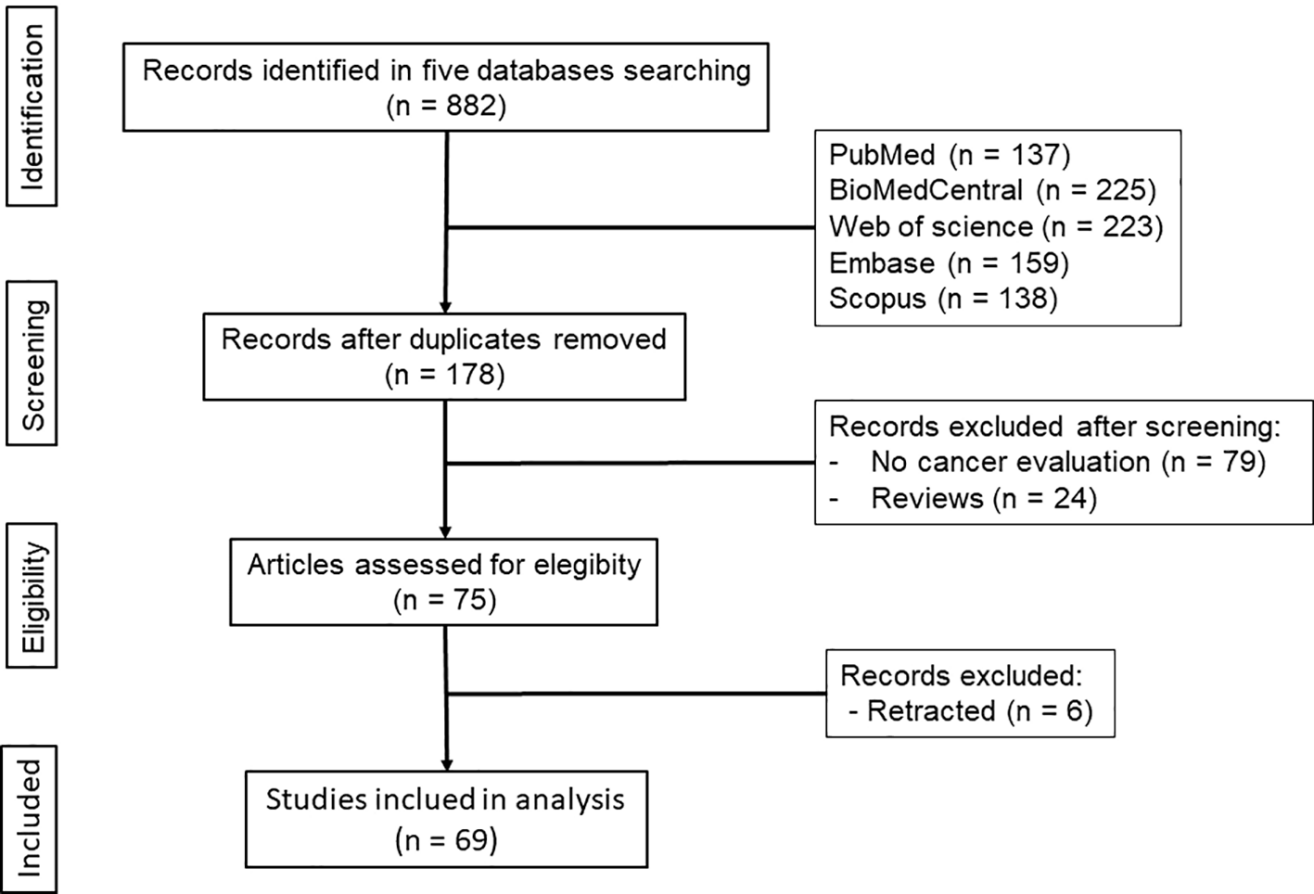
Diagram showing the search strategy and selection of eligible studies.

Systematic search in the five literature databases allowed the selection of 69 eligible studies, which were considered during the analyses (**Figure 1**).

### 3.2 circHIPK3 in cancer

circHIPK3 dysregulation was reported by 69 studies (8 downregulated and 61 upregulated study report) in 21 different cancers, being able to sponge 33 different miRNAs experimentally validated (**Table 1** and **Supplementary Table S1**). Details of the studies included in the analyses are contained in **Table 1** and **Supplementary Table S1**.

**Table 1:** circHIPK3 sponging miRNA in cancer.

Cancer	Expression	Esponged miRNA	Affected pathway or gene	Biological funtion and/or clinical significance	Ref.
Acute lymphoblastic leukemia	Up	Not reported	Not reported	Leukemic cells growth	(6)
Bladder	Down	Not reported	Not reported	Prognostic biomarker	(7)
	Down	miR-588	HPSE	Cell migration, invasion and angiogenesis, and clinicopathological features association	(8)
	Down	Not reported	Not reported	Gemcitabine resistance, and prognostic biomarker	(9)
	Down	Not reported	VCP/Beclin 1	Cell proliferation and inhibition autophagy	(10)
Breast	Up	miR-193a-5p	HMGB1/PI3K/AKT	Cell proliferation, migration, invasion and metastasis, and poor prognosis predictor	(11)
	Up	miR-326	Not reported	Cell proliferation, migration, invasion, apoptosis resistance, and tumor growth	(12)
	Up	miR-326	Not reported	Cell proliferation, migration and invasion	(13)
	Up	miR-1286	HK2	Tumor growth and chemosensitivity (Paclitaxel)	(14)
	Up	miR-124-3p	MTDH	Cell proliferation, and angiogenesis	(15)
	Up	miR-582-3p	RNF11	Trastuzumab chemoresistance	(16)
Cervical	Up	miR-338-3p	HIF-1α	Cell growth, clone formation, migration, invasion and EMT, and metastasis	(17)
	Up	miR-485-3p	FGF2	Cell proliferation, migration and invasion	(18)
Colorectal	Up	miR-637	STAT3/Bcl-2/beclin1	Oxaliplatin resistance, autophagy inhibition, and prognostic predictor for oxaliplatin-based chemotherapy	(19)
	Up	miR-1207-5p	FMNL2	Cells migration, invasion, proliferation, and metastasis	(20)
Cholangiocarcinoma	Up	miR-637	LY6E	Cell proliferation, migration, invasion and apoptosis inibition	(21)
	Up	miR-152-3p	PDK1	Cell growth, metastasis and glycolysis	(22)
Chronic myeloid leukemia	Up	miR-124	B4GALT1, NF-κB	Cell proliferation and apoptosis, and poor prognosis	(23)
Esophageal	Up	miR-599	c-MYC	Cell proliferation and invasion, and metastasis	(24)
	Up	Not reported	p53-Akt-Mdm2	Cell proliferation, migration and invasion	(25)
	Up	miR-124	Akt3	Cell proliferation, migration, epithelial-mesenchymal transition, and growth	(26)
	Up	miR-637	FASN	Cell proliferation, colony formation, migration and invasion, and tumor growth	(27)
Gallbladder	Up	miR-124-3p	ROCK1-CDK6	Cell survival and proliferation	(28)
Gastric	Up	Not reported	Not reported	Field effect biomarker	(29)
	Up	Not reported	Not reported	Biomarker	(30)
	Down	Not reported	Not reported	Prognostic biomarker	(31)
	Up	Not reported	Wnt/β-catenin	Cell proliferation and migration, and poor prognosis	(32)
	Up	miR-124; miR-29b	COL1A1/COL4A1/CDK6	Cell proliferation, T stage association, and biomarker of Ming’s histological classifcation	(33)
	Up	miR-653-5p; miR-338-3p	NRP1	Cell invasion and migration, long-term hypoxic microenvironment and prognostic biomarker	(34)
	Up	miR-876-5p	PIK3R1	Cell proliferation, migration, invasion and glutaminolysis capacities	(35)
	Up	miR-637	AKT1	Cell viability, proliferation, migration, and invasion	(36)
	Up	miR-508-3p	Bcl2/beclin1/SLC7A11	Inibition of autophagy and ferroptosis, and cisplatin resistance	(37)
Glioma	Up	miR-124-3p	STAT3	Cell proliferation, invasion and migration	(38)
	Up	miR-654	IGF2BP3	Cell proliferation and invasion, tumor propagation, and poor prognosis	(39)
	Up	miR-124	CCND2	Cell proliferation, migration and invasion, and poor prognosis	(40)
	Up	miR-524-5p	KIF2A-PI3K/AKT	Cell proliferation and metastasis, and chemosensitivity (Temozolomide)	(41)
	Up	miR-421	ZIC5	Cell progression and chemoresistance (Temozolomide)	(42)
Hepatocellular carcinoma	Up	Not reported	Not reported	Cell proliferation	(5)
	Up	miR-124	AQP3	Cell proliferation and migration	(43)
	Up	miR-338-3p	ZEB2	Cell migration, invasion, and metastasis	(44)
	Up	miR-582-3p	DLX2	Cell proliferation, migration and invasion and apoptosis inibition	(45)
	Up	miR-124-3p; miR-4524-5p	MRP4	Cell proliferation	(46)
	Up	miR-124; miR-506-5p	PDK2	Cell migration and invasion, and clinical characteristics association	(47)
Lung	Up	miR-124	SphK1, STAT3, CDK4	Cell survival and proliferation	(48)
	Up	miR-124-3p	STAT3-PRKAA/AMPKa	Autophagy regulation, and prognostic factor	(49)
	Up	miR-107	BDNF	Cell proliferation and metastasis	(50)
	Up	miR-149	FOXM1	Cell proliferation and invasion, and tumorigenesis and metastasis	(51)
	Up	miR-377-3p	PD-L1	Tumor progression and predict poor prognosis	(52)
	Up	miR-637	Not reported	Biomarker for lung cancer	(53)
Melanoma	Up	miR-215-5p	YY1	Cell proliferation, and apoptosis inhibition	(54)
Nasopharyngeal carcinoma	Up	miR-4288	ELF3	Cell proliferation, migration and invasion, and prognostic marker	(55)
Oral squamous cell carcinoma	Up	miR-124	Not reported	Cell proliferation, and prognostic marker	(56)
	Up	miR-381-3p	YAP1	Cell proliferation, invasion, migration and apoptosis inibition, and tumor growth	(57)
	Up	miR-637	NUPR1/PI3K/AKT	Cell proliferation, metastasis, and EMT	(58)
Osteosarcoma	Down	Not reported	Not reported	Cell proliferation, migration and invasion, poor prognosis, and diagnosis biomarker	(59)
	Up	miR-637	HDAC4	Cell proliferation, migration and invasion, prognostic marker, and diagnosis biomarker	(60)
Ovarian	Up	Not reported	Not reported	Poor prognosis	(61)
	Up	miR-7	VEGF	Cell proliferation and apoptosis	(62)
	Down	Not reported	Not reported	Cell proliferation, migration and invasion	(63)
Pancreatic cancer	Up	miR-330-5p	RASSF1	Cell proliferation, invasion, migration, apoptosis, EMT, and gemcitabine resistance and poor prognosis	(64)
	Up	miR-1179	RHPN2	Cell proliferation, migration, invasion, and angiogenesis	(65)
Prostate	Up	miR-338-3p	ADAM17	Cell proliferation, and invasion	(66)
	Up	miR-193a-3p	MCL1	Cell proliferation, migration, and invasion, and poor prognosis	(67)
	Up	miR-338-3p	Cdc25B; Cdc2	Cell viability, proliferation and apoptosis inibition	(68)
	Up	miR-448	MTDH	Cell migration, proliferation and invasion, and tumor growth	(69)
	Up	miR-212	BMI1	Cell proliferation, metastasis and tumorigenesis	(70)
Renal carcinoma	Down	miR-637	Not reported	Cell proliferation, migration and invasion	(71)
	Up	miR-485-3p	EMT	Cell proliferation, apoptosis inhibition and metastasis, and prognostic marker	(72)
	Up	miR-508-3p	CXCL13	Cell proliferation and metastasis, and poor clinicopathological features	(73)

Ref., References; Up, Upregulation; Down, Downregulation.

Eight studies found this circRNA downregulated (7–10, 31, 59, 63, 71). However, some of them used paraffin preserved tissue (31), samples from patients receiving chemotherapy (59), controls samples from patients with another type of tumor (63), and normal samples collected very close to the tumor (e.g., normal tissues ≥ 1cm far away to tumor) (71). These factors may cause biases for the evaluation of gene expression. For instance, studies demonstrated that long RNAs are more susceptible to degradation than small RNAs ones in paraffin preserved samples (78, 79). In addition, samples collected adjacent to the tumor (and used as normal tissue) can be influenced by the tumor microenvironment and have their gene expression profile altered when compared to a truly healthy tissue (field cancerization phenomenon) (79, 80).

The interaction of circHIPK3 and six sponged miRNAs have been reported in more than one cancer type, for example, circHIPK3/miR-124-3p was reported in 13 different studies, circHIPK3/miR-637 in eight studies, circHIPK3/miR-338-3p in four studies, and circHIPK3/miR-326, circHIPK3/miR-485-3p circHIPK3/miR-508-3p and circHIPK3/miR-582-3p in two studies each (**Table 1**; **Supplementary Table S1**).

The prognostic and diagnosis value of circHIPK3 in different cancers has been investigated. For example, low expression levels are correlated with high pathological grade, risk of progression, lymph node metastasis and gemcitabine resistance in bladder cancer (7, 9). On the other hand, high expression levels are correlated with a worse prognosis (11), as well as paclitaxel (14) and trastuzumab (16) resistance in breast cancer. In colorectal cancer, its upregulation is correlated with tumor size, lymph node metastasis, distant metastasis, recurrence, poor survival and oxaliplatin resistance (19). It is correlated to poor overall survival rate in cholangiocarcinoma (23), chronic myeloid leukemia (24) and ovarian cancer (62). In osteosarcoma, it is correlated to shorter overall survival times and poor prognosis, serving as a biomarker (AUC = 0.783; 0.875) (60, 61). In lung cancer, it has been suggested as a biomarker (AUC = 0.897) (54). In gastric cancer, it is correlated to poor overall survival rate (33), T stage, Ming’s classification and infiltrative type (34), and cisplatin resistance (37). In glioma, it is correlated with unfavorable prognosis (40) and temozolomide resistance (42, 43). In nasopharyngeal carcinoma, it is correlated to lower overall survival and distant metastasis‐free survival rate (56). In oral cancer, it is correlated to distant metastasis, higher tumor staging and shorter survival (59). In pancreatic cancer, it is correlated to gemcitabine resistance (65). In renal cancer, it is correlated to lymph node metastasis and shorter survival rates (73, 74). Therefore, circHIPK3 appears to be potentially useful as a prognostic and diagnostic marker.

Considering ethnic populations, the dysregulation of this circRNA in cancer has been reported mainly in the Asian population, however, it was found in the European population and in the mixed Brazilian population, in South America (**Supplementary Table S1**).

### 3.3 Enrichment analysis

**Figure 2:**
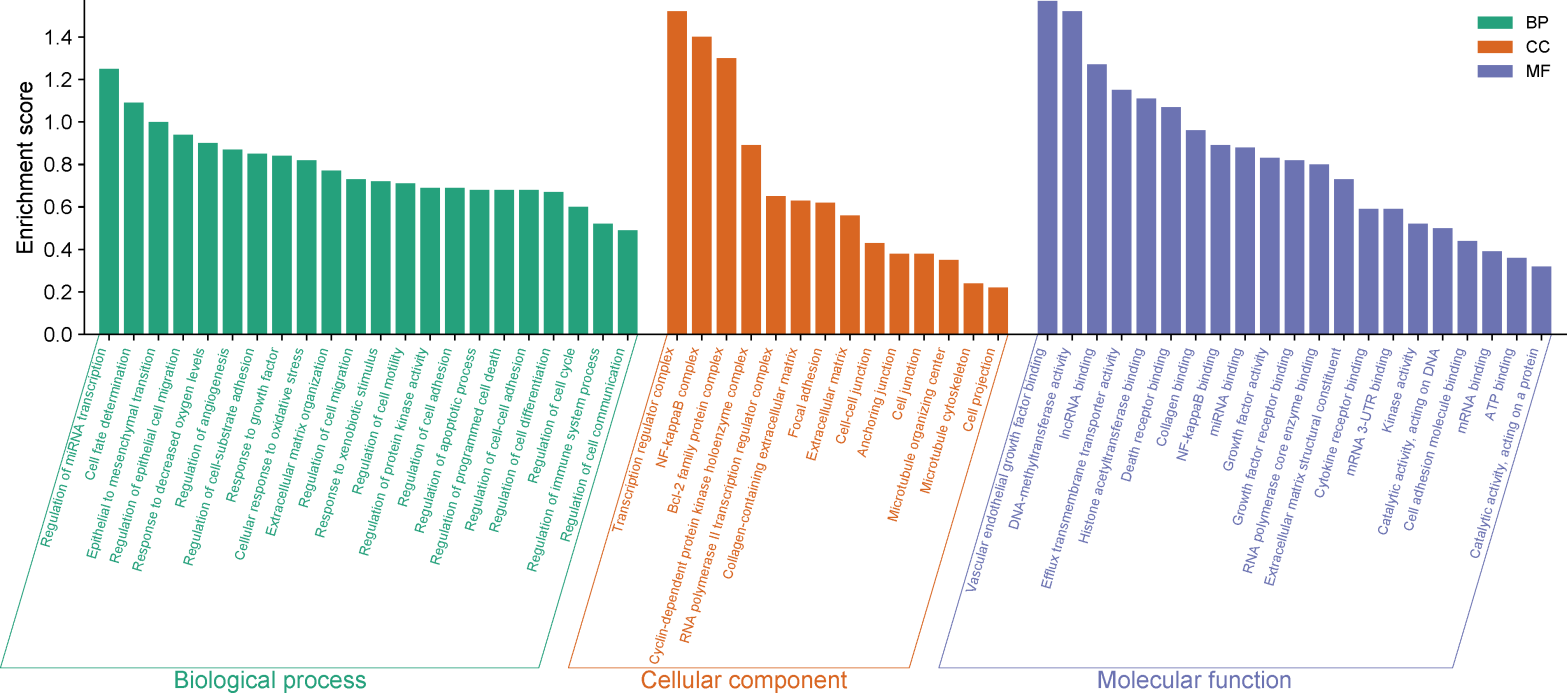
Target genes enrichment in Gene Ontology.

Except for miR-4524-5p, the other 33 miRNAs can regulate a total of 399 genes experimentally validated by strong evidences (**Supplementary Table S2**). Enrichment analysis of the 399 target genes in Gene Ontology showed that many of them were enriched in biological processes, cellular structural composition and molecular functions related to cell differentiation, fate, adhesion, motility, migration, growth and death. Furthermore, many have been related to epithelial to mesenchymal transition, inflammation, immunity, gene expression, RNA processing and interaction to messenger and non-coding RNAs (**Figure 2**; **Supplementary Table S3**).

**Figure 3:**
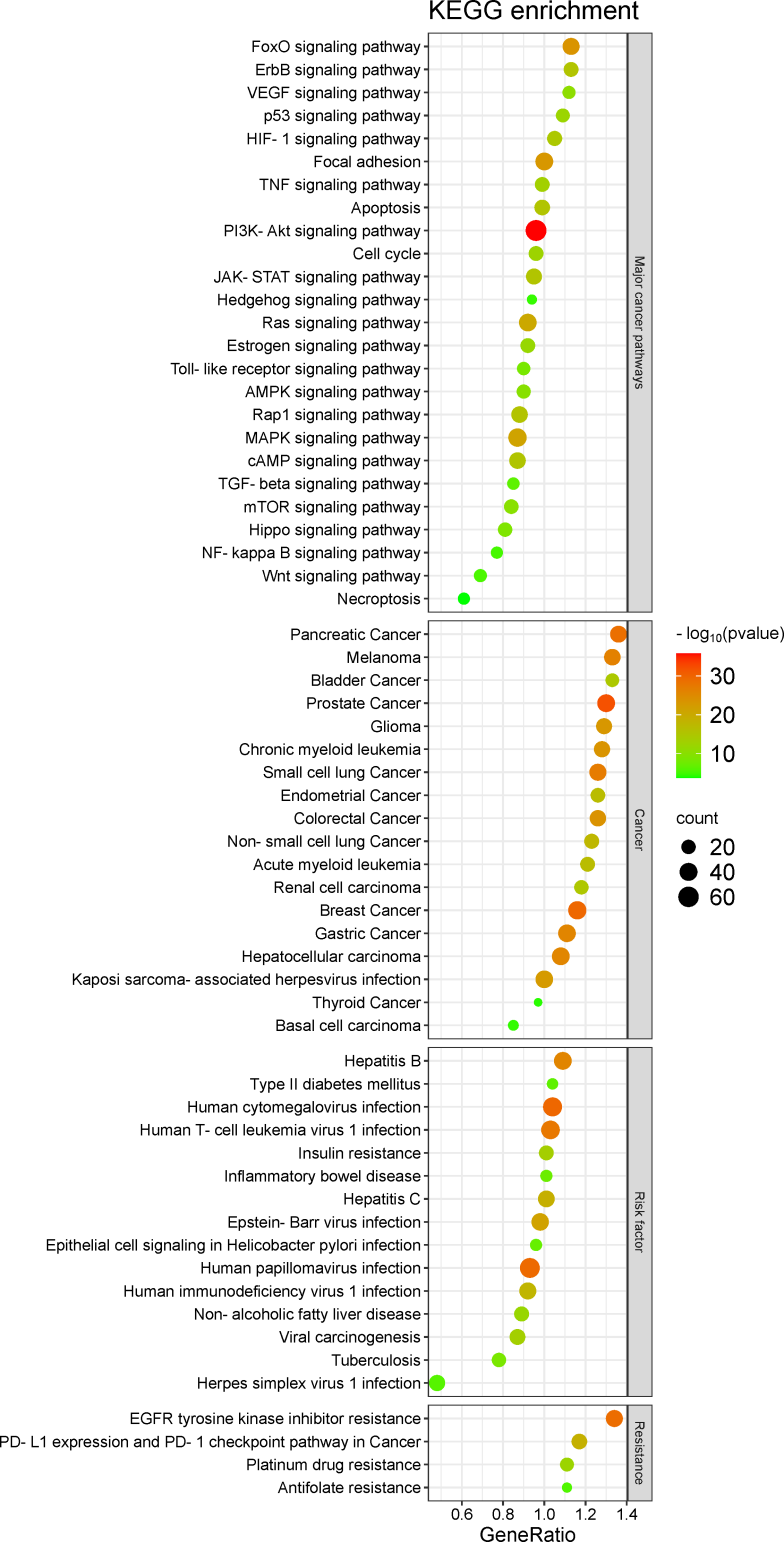
Target genes enrichment in KEGG pathways.

In KEGG pathways, gene enrichment was organized into four distinct classes, such as 1) major cancer-related biological pathways, 2) cancer pathways, 3) risk factors-related pathways for cancer development, and 4) chemoresistance-related pathways in cancer (**Figure 3**). For example, of the 25 highlighted pathways from the “major cancer-related biological pathways” class, we highlight the PI3K-Akt signaling pathway [hsa04151] which has 64 enriched target genes (**Supplementary Table S4**). Many target genes were also enriched in 18 different cancer pathways, 15 pathways associated to risk factors for the development of different types of cancer, and at least four pathways directly associated to chemoresistance (**Figure 3**; **Supplementary Table S4**).

Considering pathways associated to anti-cancer agent resistance treatments, some target genes were enrichment in antifolate resistance (8 target genes), platinum drug resistance (18 target genes), PD-L1 expression and PD-1 checkpoint (26 target genes), and EGFR tyrosine kinase inhibitor resistance (34 target genes) pathways (**Supplementary Table S4**). The target genes such as *ABCC1* (regulated by miR-7-5p) and *ABCC4* (target of miR-124-3p), *ABCG2* (target of miR-212-3p) and *GJA1* (target of miR-381-3p) are involved in “efflux transmembrane transporter activity” (GO:0015562; four target genes) (**Supplementary Table S3**) and platinum drug resistance (hsa01524; 26 target genes; (**Supplementary Table S4**). In addition, some of these miRNAs also regulate solute transport genes, such as *SLC7A5* (target of miR-7-5p), *SLC16A1* (target of miR-124-3p), and *SLC40A1* (target of miR-485-3p) (**Supplementary Table S2**). These genes are involved in transmembrane transport activity and chemoresistance (81–83).

We observed that *RAD51* (recombinase) and *MGMT* (O-6-methylguanine-DNA methyltransferase) genes, responsible for repairing DNA damage caused by chemotherapeutic alkylating and platinum derivatives, can be regulated by miR-107 and miR-124-3p, respectively (**Supplementary Table S2**).

**Figure 4:**
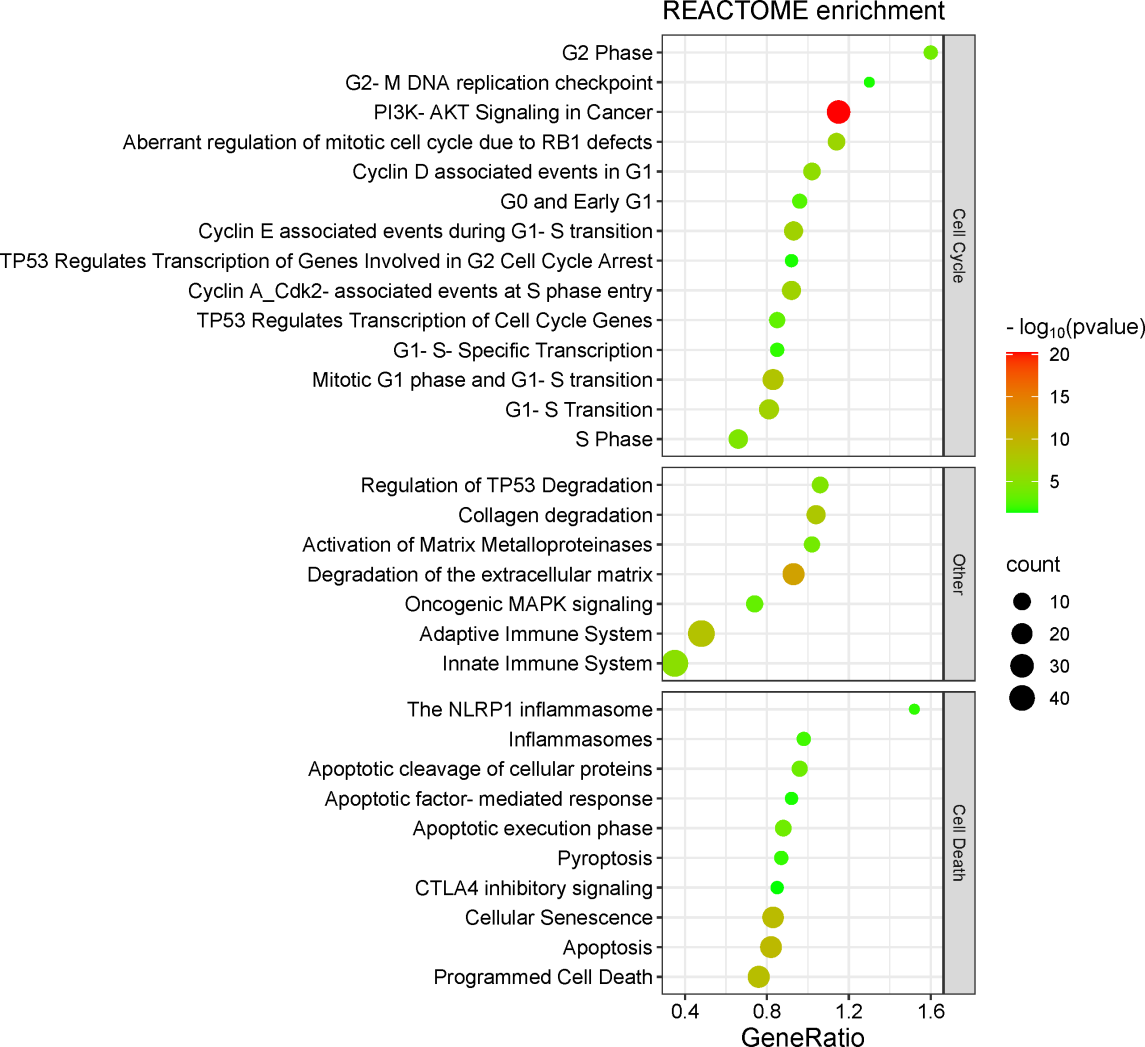
Target genes enrichment in REACTOME pathways.

In REACTOME pathways, gene target enrichment was organized into three classes, such as 1) cell cycle-related pathways, 2) “other” – pathways related to immune system, TP53 modulation and extracellular matrix remodeling (important to support cancer invasion and metastasis), and 3) cell death-related pathways (**Figure 4**; **Supplementary Table S5**).

Seventy-six target genes can be regulated by at least two different miRNAs, of which we highlighted oncogenes and tumor suppressors that can be regulated by at least three (*BCL2*, *CDH2*, *CCND1*, *CD274*, *DNMT3B*, *DNMT1*, *GRN*, *MYC, MMP9, SP1*, *PI3KR3*, *KLF4* and *IGF1R*), and four (*CDK6*, *CD151* and *PTEN*) different sponged miRNAs (**Supplementary Table S6**).

**Figure 5:**
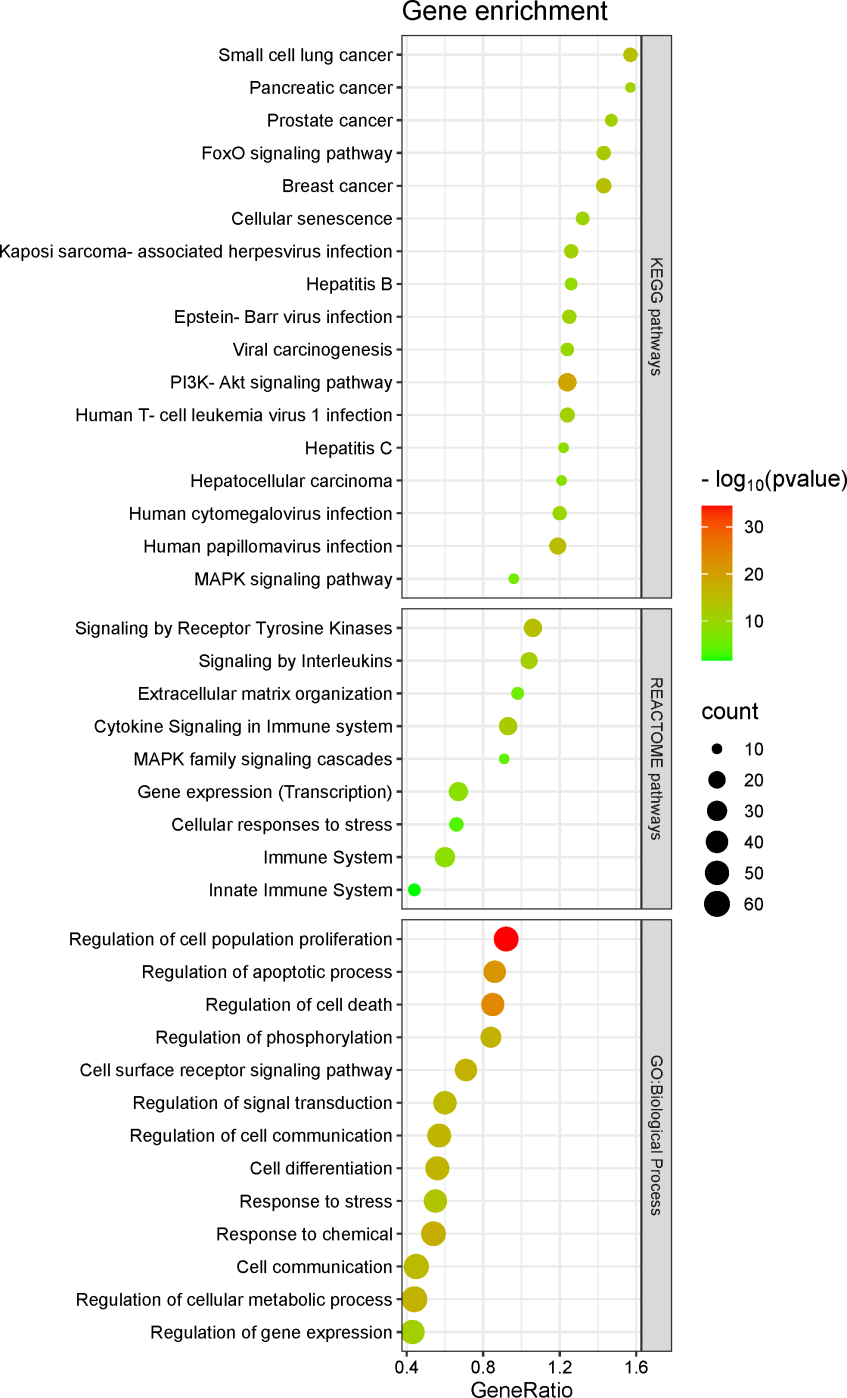
Seventy-six target genes enrichment.

To better understand the importance of these 76 target genes, we performed their gene enrichment and selected biological processes from Gene Ontology that presented a number ≥40 enriched target genes, and in KEGG and REACTOME pathways with a number ≥10 enriched target genes (**Figure 5**; **Supplementary Table S7**). These target genes were enriched in functions related to cell control (e.g., differentiation, adhesion, motility, migration, growth and death), to epithelial to mesenchymal transition, inflammation, immunity, and oncogenic viral infection (**Figure 5**).

### 3.4 RBPs enrichment

**Figure 6:**
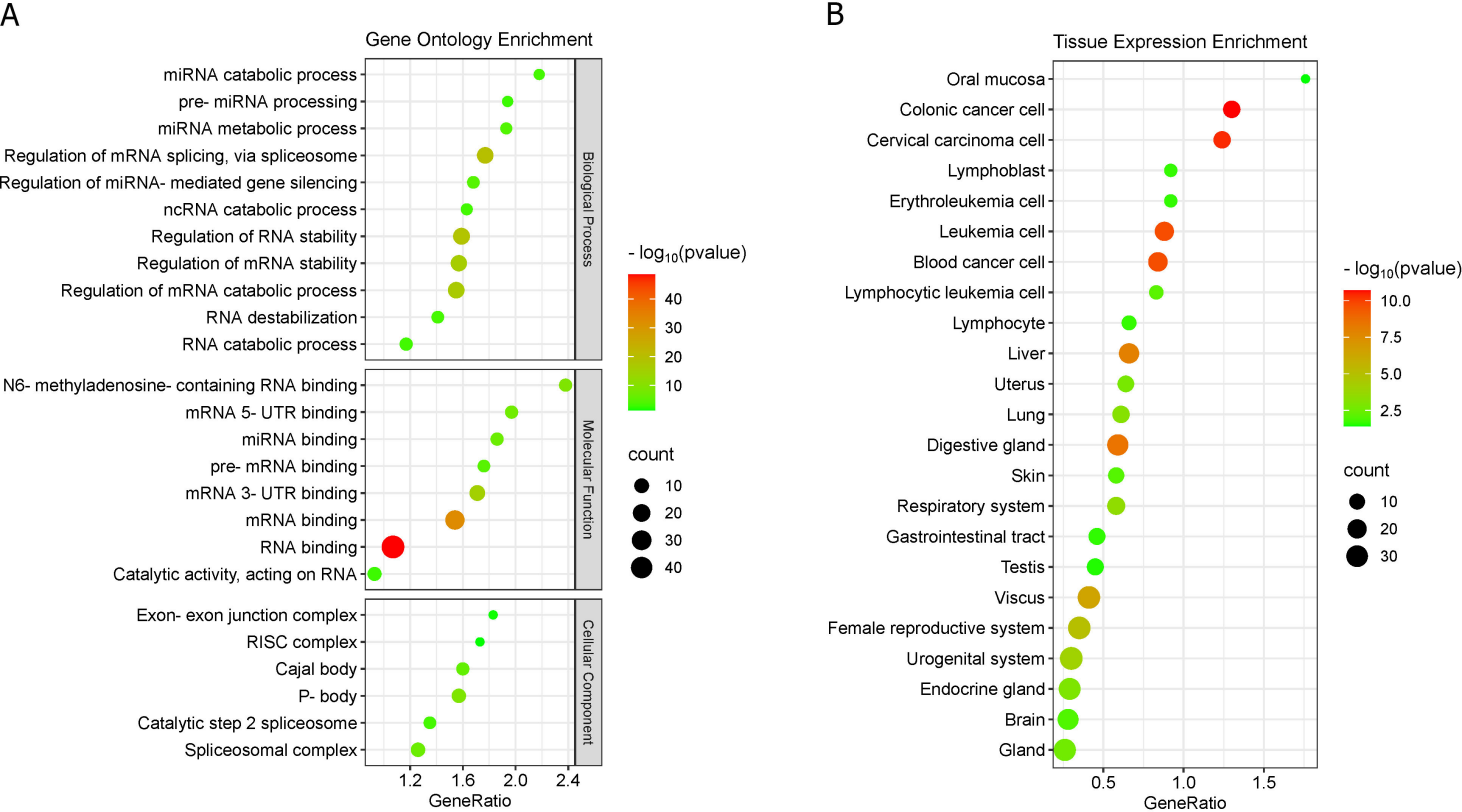
RBPs enrichment. **(A)** RBPs enrichment in Gene Ontology. **(B)** RBPs enrichment in TISSUE expression database.

Gene enrichment (molecular process) of the 50 RBPs that have binding sites in circHIPK3 (3, 30), showed that these proteins participate of biological processes, cellular structural composition and molecular functions related mainly to RNA metabolism and regulation of gene expression (**Figure 6A**). We highlight the DDX54, EIF4A3, FMR1, IGF2BP1, IGF2BP2, LIN28B and MOV10, since they have more than 10 circHIPK3 binding sites (**Supplementary Table S8**).

We highlight the molecular function of six RBPs (HNRNPA2B1, IGF2BP1, IGF2BP2, IGF2BP3, YTHDF1, and YTHDC1) related to N^6^-methyladenosine-(m^6^A)-containing RNA binding [GO:1990247] (**Figure 6A**; **Supplementary Table S8**). The m^6^A markers are important degradation signals for several classes of RNA, which are recognized by m^6^A-binding proteins (“readers”), such as HNRNPA2B1, IGF2BP1/2/3, YTHDF1/2/3 and YTHDC1/2 (84).

Some RBPs compose the Cajal body (DKC1, FBL, FMR1, HNRNPA2B1, NOP58, and SMNDC1) and P-body (CAPRIN1, ELAVL1, IGF2BP1, IGF2BP2, IGF2BP3, LIN28A, MOV10, UPF1, and YTHDF1) organelle-like structures (**Figure 6A**; **Supplementary Table S8**). These non-membrane organelle-like structures are associated to RNA storage and decomposition, and cancer (85, 86). Additionally, we found that many of these RBPs are abundantly expressed in cancer cells and in human tissues (**Figure 6B**).

## 4 Discussion

In recent years, studies have demonstrated and reinforced the importance of circRNAs in cancer pathogenesis, and the clinical impact of mRNA-target-miRNA/circHIPK3/RBP interaction network in cell biological processes (3).

Sixty-seven studies in cancer report the dysregulation of circHIPK3 in cancer, and that it may interfere in 33 miRNAs activity by sponging them (5, 8, 11–24, 26–28, 33–58, 60, 62, 64–73). These studies show that the homeostasis break of circHIPK3/33-miRNAs/mRNA-targets results in disturbance of pathways that lead to the loss of cellular control, causing proliferation, migration, invasion, evasion of apoptosis and autophagy, EMT, metastasis and chemoresistance, and its overexpression was associated to poor prognosis (5–73).

Indeed, our functional analyses show that many of the target genes of sponged miRNAs can modulate pathways that control these biological and cellular processes. For example, in our analyses PI3K-Akt signaling pathway was highlighted due to the involvement of the large number of target genes identified, of which we highlight the *AKT1*, *AKT3*, *BDNF*, *CCND2*, *CDK4*, *CDK6*, *COL4A1* and *PIK3R1*. These genes are directly affected by circHIPK3 in gastric cancer [miR-124/miR-29b/*COL4A1*/*CDK6* (33), miR-876-5p/*PIK3R1* (35) and miR-637/*AKT1* (36)], gallbladder cancer [miR-124/*CDK6* (28)], breast cancer [circHIPK3/*AKT* (11)], glioma [miR-124/*CCND2* (40)], lung cancer [miR-124/*CDK4* (48) and miR-107/*BDNF* (50)] and oral squamous cell carcinoma [miR-637/*AKT* (58)]. PI3K-Akt is a highly conserved and extremely important pathway for cellular homeostasis, as it is responsible for promotes cell proliferation, survival, metabolism, growth, apoptosis and angiogenesis in response to extracellular signals (87).

Dysregulation of circHIPK3/miR-124-3p has been reported in breast (15), chronic myeloid leukemia (23), esophageal (26), gallbladder (28), gastric (33), glioma (38, 40), hepatocellular carcinoma (43, 46, 47), lung (48, 49), and oral squamous cell carcinoma (56) cancers, demonstrating it to be an important interaction for cancer development. Indeed, miR-124-3p is a tumor suppressor miRNA in cancers and is able to control PI3K-Akt signaling pathway (88). circHIPK3/miR-637 has been reported in colorectal (19), cholangiocarcinoma (21), esophageal (27), gastric (36), lung (53), oral squamous cell carcinoma (58), osteosarcoma (60), and renal carcinoma (71) cancers. miR-637 is able to modulating the Jak/STAT3, Wnt/β-catenin, and PI3K/Akt signaling pathways; it is downregulated in cancer and is associated to larger tumors and later tumor node metastasis staging in cancer patients (89). circHIPK3/miR-338-3p has been reported in cervical (17), gastric (34), hepatocellular carcinoma (44), and prostate (66, 68) cancers. miR-338-3p is downregulated in cancer and has an important role during tumor progression by modulation of Wnt, MAPK, and PI3K/Akt signaling pathways (90). Interestingly, MAPK, Jak/STAT3, Wnt/β-catenin, and PI3K/Akt signaling pathways were abundantly enriched by the target genes analyzed (**Figure 2**). Therefore, these evidence together suggest that circHIPK3 acts in the functional silencing of these tumor suppressor miRNAs, acting as an onco-cirRNA and favoring cancer development.

circHIPK3 functional role and expression in cancer remains dubious, for example, a massive number of studies demonstrate that circHIPK3 is upregulated (5, 6, 11–30, 32–58, 60–62, 64–70, 72, 73), while few studies it is downregulated (7–10, 31, 59, 63, 71). This circRNA was found downregulated in bladder cancer (7–10), and its expression is even lower in muscle invasive bladder cancer (e.g., tumor invades the lamina propria and detrusor muscle) when compared with non-muscle invasive bladder cancer (e.g., tumor limited to the urothelium) (91). An interesting aspect associated to the normal bladder physiology is the existence of a urinary microenvironment permeated by the presence of considerable amounts of hydrogen peroxide (H_2_O_2_), a reactive oxygen species (ROS) (92). It was also identified that in cancer patients (e.g., breast, cervical, esophagus and laryngeal carcinoma) the urine concentration of H_2_O_2_ is two- to three-fold higher than in healthy people (92). In human bladder cancer (urothelial carcinoma), was showed that elevated levels of ROS induced by Nox4 enzyme (a H_2_O_2_-generator enzyme) are required for tumor initiation and progression (93). It was also observed that H_2_O_2_ can induce a metastatic phenotype in bladder cancer cells (94). In mouse model, was observed that healthy urothelial cell produce H_2_O_2_ in response to calcium signaling (95). Curiously, H_2_O_2_ is able to put down circHIPK3 expression in osteoblast (96), cardiomyocyte (97) and lens epithelial (98) cell human. Therefore, the circHIPK3 downregulation observed in bladder cancer may be a consequence of the normal and tumoral H_2_O_2_-elevated milieu characteristic of this organ.

Many target genes were enriched in pathways related to risk factors associated to the cancer, such as type II diabetes mellitus, inflammatory bowel disease and tuberculosis (**Figure 2**). Indeed, circHIPK3 contributes to hyperglycemia and insulin resistance by disturbing the miR-192-5p/*FOXO1* axis homeostasis (99) and is upregulated in type II diabetes patients (100), and it was found upregulated in acute pancreatitis (101), an important risk factor for the pancreatic cancer development (102).

This circRNA was observed to be downregulated in Crohn’s disease and ulcerative colitis (103), conditions that lead to atrophy of the intestinal epithelium (104) and favors the onset of colorectal cancer (105). However, its expression increases the proliferation of intestinal cells by sponging miR-29b, contributing to the renewal of the intestinal mucosa after injury caused by these diseases (103). Therefore, circHIPK3 acts on the proliferation of intestinal mucosa cells, maintaining their renewal and homeostasis (103), however, when dysregulated (e.g., upregulated) it can lead to colorectal cancer (19, 20). In addition, circHIPK3 can sponge miR-29b-3p and abolish its function in gastric cancer (33), so this interaction may be an important mechanism for epithelial mucosal proliferation in gastrointestinal cancers. Interestingly, the circHIPK3 downregulation observed in Crohn’s disease and ulcerative colitis (91), may be a consequence of the high levels of H_2_O_2_ produced during the development of these diseases (106, 107).

In lung cancer, circHIPK3 is overexpressed (48–52). Its overexpression also induces pulmonary fibrosis by interfering with the circHIPK3/miR-30a-3p/*FOXK2* axis (108) and by inducing fibroblast-to-myofibroblast transition via regulation of circHIPK3/miR-338-3p/*SOX4*/*COLA1A1* (109), this condition is a factor in risk for lung cancer (110). Furthermore, its overexpression is related to pulmonary arterial hypertension development (111), whose prevalence is higher in patients with lung cancer (112).

circHIPK3 is upregulated in acute kidney injury and affects the activity of miR-93-5p (113), miR-124-3p (114), miR-148b-3b (114, 115) and miR-338-3p (116). Upregulation of circHIPK3 is also associated to chronic tubulointerstitial nephritis and renal tubulointerstitial fibrosis by regulating miR-30a/profibrotic-proteins axis (117). In renal tubular epithelial cell, its upregulation induces cell proliferation and inhibition of the apoptosis by miR-326 and miR-487a-3p modulation (118). It was also found overexpressed in podocytes subjected to injury caused by high-glucose concentrations (119), this injury is a common condition in diabetes mellitus. These conditions are an important risk factor for the development of kidney cancer (120), since it is also overexpressed in this type of cancer (72, 73). Interestingly, circHIPK3 regulates the miR-124-3p (15, 23, 26, 28, 33, 38, 40, 43, 46–49, 56), miR-326 (12, 13) and miR-338-3p (17, 34, 44, 66, 68) activity in cancer.

circHIPK3 plays an important role in resistance to many anti-cancer agents. For example, its overexpression favors oxaplatin resistance, apoptosis and autophagy inhibition in colorectal cancer by abolishing the regulatory function of miR-637 on STAT3/Bcl-2/beclin1 signaling pathway (19). In gastric cancer, it promotes cisplatin resistance as a consequence of apoptosis, autophagy, and ferroptosis inhibition by abolishing the regulatory power of miR-508-3p on the ↑Bcl-2/beclin1/SLC7A11 axis (37). The effect of circHIPK3 on gemcitabine resistance is attributed to its negative regulatory role on miR-330-5p which enables RASSF1 activity, favoring proliferation, invasiveness, migration, EMT, and apoptosis inhibition in pancreatic cancer (64). In breast cancer, paclitaxel resistance occurs due to circHIPK3/miR-1286/HK2 modulation and consequent cell cycle progression and apoptosis inhibition (14); and trastuzumab resistance occurs through the transmission of circHIPK3 via exosomes and miR-582-3p/RNF11 axis regulation, which promotes cell proliferation, invasion, and apoptosis inhibition (16). Temozolomide resistance arises as a consequence of the modulation of the cell concentration (IC_50_) of temozolomide and apoptosis inhibition by circHIPK3/miR-524-5p/KIF2A (41) and exosomal-circHIPK3/miR-421/ZIC5 (42) axis regulation in glioblastoma. Therefore, although the details of the molecular mechanisms leading to circHIPK3-mediated chemoresistance are not fully understood, interference by this circRNA in distinct and complex pathways resulting in the inhibition of cell death may be a key mechanism in this process.

In bladder cancer, it has been observed that circHIPK3 downregulation favors gemcitabine resistance (9), although the underlying mechanism remains unknown. *NXPH4* overexpression has been shown to induce gemcitabine resistance in bladder cancer by increasing ROS and glycolysis levels (121). Since ROS is a known factor that can downregulate circHIPK3 (96–98), this suggests a potential link. Furthermore, low expression levels of LOXL4 and SRSF2 – genes regulated by miR-29b-3p and miR-193a-3p (see **Supplementary Table S2**) – have been associated with inhibited apoptosis and enhanced multi-drug resistance in bladder cancer (122). Therefore, the ROS-induced *NXPH4*/↓circHIPK3/↑miR-29b-3p/↑miR-193a-3p/↓LOXL4*/*↓SRSF2 axis may represent a potential mechanism through which circHIPK3 downregulation contributes to gemcitabine resistance in bladder cancer.

Several target genes were enriched in the platinum drug resistance pathway (hsa01524), including the *BCL2* oncogene, which can be regulated by at least three miRNAs sponged by circHIPK3 (miR-7-5p, miR-29b-3p, and miR-448). In fact, circHIPK3 overexpression can affect the activity this gene and induce resistance to oxaliplatin in colorectal cancer (19) and to cisplatin in gastric cancer (37). Genes belonging to ATP-binding cassette (ABC) transportes, such as *ABCC1* (regulated by miR-7-5p), *ABCC4* (regulated by miR-124-3p), *ABCG2* (regulated by miR-212-3p), and *GJA1* (regulated by miR-381-3p) were enriched in efflux transmembrane transporter activity (GO:0015562). In cancer cells that have multidrug resistance generally upregulate these genes, which are associated with the efflux of chemotherapeutics out of cells and, therefore, decrease chemosensitivity to anticancer drugs (82, 83). In addition, *SLC7A5* (regulated by miR-7-5p), *SLC16A1* (regulated by miR-124-3p), and *SLC40A1* (regulated by miR-485-3p) genes, belong to the transmembrane solute transport (*SLC*) and are involved in chemoresistance in many of the cancers studied here (81). Interestingly, circHIPK3/miR508-3p interaction can modulate SLC7A11 activity and induce cisplatin resistance in gastric cancer (37). Therefore, the modulation of these genes by sponging these miRNAs could be another important mechanism associated to chemoresistance involving circHIPK3.

miR-124-3p can regulate the *MGMT* gene, responsible for repairing DNA damage caused by chemotherapeutic alkylating and alkylating-like agents (e.g., gemcitabine, temozolomide and platinum-based drugs). Indeed, *MGMT* overexpression can lead to apoptosis escape induced by alkylating agents (123). For example, *MGMT* overexpression induces resistance to gemcitabine in pancreatic cancer (124), to cisplatin in colorectal cancer (125) and to temozolomide in glioma (126, 127) and estrogen receptor positive breast cancer (128). Thus, circHIPK3/miR-124-3p/MGMT may modulate resistance to multi-drugs in these cancers. On the other hand, the *RAD51* recombinase gene (which encodes a protein that is essential for repairing damaged DNA) is regulated by miR-107. Interestingly, overexpression of this protein is associated with gemcitabine resistance in lung (129) and contributes to chemotherapy-induced damage and the destabilization of genetic material in cancer cells (130). Therefore, the circHIPK3/miR-107/*RAD51* axis may be another mechanism that induces chemotherapy resistance in cancer.

N6-methyladenosine (m^6^A) is a modification that occurs internally in long RNAs (e.g., circRNA) that are recognized by “readers” (e.g., *IGF2BPs*), and when deregulated they can lead to cancer (83). Interestingly, circHIPK3 appears to be a peculiar molecule, because despite not having any m^6^A modification in its structure that signals for the binding of readers such as *IGF2BPs* (131), it surprisingly maintains many binding sites for RNA-binding proteins (RBPs), including for *IGF2BPs* group (**Supplementary Table S8**). *IGF2BP1*, *IGF2BP2* and *IGF2BP3* are dysregulated in many tumor types (84), being associated with chemoresistance in glioblastoma (132), ovarian (133) and colorectal (134) cancer. In fact, circHIPK3 sequesters IGF2BP2 allowing the target gene of this RBP, *STAT3* oncogene (responsible for controlling cell proliferation and survival), to be expressed. Therefore, circHIPK3 would act as competing endogenous RNA (ceRNA) for *IGF2BP2* (92). It has also been reported that this circRNA can act as a scaffold for E3 ubequitin ligase (135). Interestingly, the existence of m^6^A and recognition by *YTHDF2* “readers” is a pathway by which circRNA are degraded (136), so the absence of m^6^A in circHIPK3 may have conferred more stability to this complex molecule.

FMR1 is an interesting RBP, since it is associated with cancer cell growth, metastasis, EMT, apoptosis, and angiogenesis (137), and has 35 binding sites in circHIPK3 (**Supplementary Table S8**). Recent evidence demonstrates that circHIPK3 binds to *BRCA1* messenger RNA, conferring stability and protection against FRM1 protein (a negative regulator of *BRCA1*), allowing the effective expression of this oncogene. Therefore, circHIPK3 acts as ceRNA by binding to *BRCA1* messenger RNA using the same binding site used by FRM1, and this interaction causes chemoresistance to DNA-damaging drugs (138).

Other RBPs such as DDXR54, EIF4A, LIN28B and MOV10 have multiple binding sites for circHIPK3. These RBPs are important for stability and functional role of circRNAs in several types of cancers. For example, *DDXR54* binding to long non-coding RNAs and confers stability to genes that contribute to cell growth in gastric cancer (139) and that facilitate stemness and EMT of osteosarcoma cells (140). *EIF4A* modulates the expression of some circRNAs by flanking specific sequences of pre-mRNA of its target gene, contributing to cisplatin resistance in bladder cancer (141). circHIPK3/miR-107/*LIN28B* axis may be a mechanism of chemoresistance in gastric (3) and ovarian (133) cancers. *MOV10* can bind to circ-DICER1 and modulate the cell viability, migration, and angiogenesis in glioma (142). Therefore, the interaction of circHIPK3 with these RBPs may participate in important mechanisms for the development of cancer and chemoresistance.

Our strategy of combining experimentally validated public data and robust bioinformatics tools for functional analysis made it possible to identify alternative routes that explain the diversity of functions exerted by circHIPK3 and its implication in multidrug resistance. A limitation of this study is that we did not experimentally test the axes modulated by this circRNA suggested here, therefore, future experimental validations of these pathways are necessary. We highlight the need for more studies on gallbladder cancer, leukemia, melanoma, nasopharyngeal carcinoma and thyroid cancer to strengthen the amount of evidence, as well as expanding the representation of other ethical populations not yet studied regarding the expression of this circRNA. Finally, our results undoubtedly open new perspectives towards understanding how circHIPK3 can exert a modulating role on establishment, progression and chemoresistance in different cancers.

## 5 Conclusions

In this study, we observed that circHIPK3 is dysregulated and that it can regulate 33 miRNAs in different types of cancer, whose target genes control important processes and biological pathways for the cancer establishment and maintenance. circHIPK3/miR-124-3p/miR-637/miR-338-3p are the most well documented interactions in various cancers types, and can control MAPK, Jak/STAT3, Wnt/β-catenin, and PI3K/Akt signaling pathways, and may be important for support the initiation and establishment of the cancer. miR-7-5p/*ABCC1*/*SLC7A5*, miR-107/*RAD51*, miR-124-3p/*ABCC4*/*SLC16A1*/*MGMT*, miR-212-3p/*ABCG2*, miR-381-3p/*GJA1* and miR-485-3p/*SLC40A1* may modulate pathways that confer chemoresistance to cancer cells. circHIPK3 contains multiple sites for the same RBPs (e.g., DDX54, EIF4A3, FMR1, IGF2BP1, IGF2BP2, LIN28B and MOV10), many of which are involved in chemoresistance and organelle-like structures, such as Cajal body and P-body which are associated with cancer. Additionally, circHIPK3 is upregulated in cancer in general acting as an onco-circRNA, except in bladder cancer, which has a likely TS-circRNA function due to the microenvironment with large amounts of H_2_O_2_ present in this organ. Therefore, circHIPK3 is a complex and multifunctional molecule that favors the establishment, progression and chemoresistance of cancers, making it an interesting molecule with a potential therapeutic target.

## Data Availability

The original contributions presented in the study are included in the article/**Supplementary Material**, further inquiries can be directed to the corresponding author/s.
